# Denitrifying metabolism of the methylotrophic marine bacterium *Methylophaga nitratireducenticrescens* strain JAM1

**DOI:** 10.7717/peerj.4098

**Published:** 2017-11-28

**Authors:** Florian Mauffrey, Alexandra Cucaita, Philippe Constant, Richard Villemur

**Affiliations:** 1 INRS–Institut Armand-Frappier, Laval, Québec, Canada; 2 Laboratoire de santé publique du Québec, Ste-Anne-de-Bellevue, Québec, Canada

**Keywords:** Denitrification, Marine bacterium, *Methylophaga*, Nitrous oxide, Nitrate

## Abstract

**Background:**

*Methylophaga nitratireducenticrescens* strain JAM1 is a methylotrophic, marine bacterium that was isolated from a denitrification reactor treating a closed-circuit seawater aquarium. It can sustain growth under anoxic conditions by reducing nitrate (${\mathrm{NO}}_{3}^{-}$) to nitrite (${\mathrm{NO}}_{2}^{-}$). These physiological traits are attributed to gene clusters that encode two dissimilatory nitrate reductases (Nar). Strain JAM1 also contains gene clusters encoding two nitric oxide (NO) reductases and one nitrous oxide (N_2_O) reductase, suggesting that NO and N_2_O can be reduced by strain JAM1. Here we characterized further the denitrifying activities of *M. nitratireducenticrescens* JAM1.

**Methods:**

Series of oxic and anoxic cultures of strain JAM1 were performed with N_2_O, ${\mathrm{NO}}_{3}^{-}$ or sodium nitroprusside, and growth and N_2_O, ${\mathrm{NO}}_{3}^{-}$, ${\mathrm{NO}}_{2}^{-}$ and N_2_ concentrations were measured. Ammonium (${\mathrm{NH}}_{4}^{+}$)-free cultures were also tested to assess the dynamics of N_2_O, ${\mathrm{NO}}_{3}^{-}$ and ${\mathrm{NO}}_{2}^{-}$. Isotopic labeling of N_2_O was performed in ^15^NH_4_^+^-amended cultures. Cultures with the JAM1Δ*narG1narG2* double mutant were performed to assess the involvement of the Nar systems on N_2_O production. Finally, RT-qPCR was used to measure the gene expression levels of the denitrification genes cytochrome *bc*-type nitric oxide reductase (*cnorB1* and *cnorB2*) and nitrous oxide reductase (*nosZ*), and also *nnrS* and *norR* that encode NO-sensitive regulators.

**Results:**

Strain JAM1 can reduce NO to N_2_O and N_2_O to N_2_ and can sustain growth under anoxic conditions by reducing N_2_O as the sole electron acceptor. Although strain JAM1 lacks a gene encoding a dissimilatory ${\mathrm{NO}}_{2}^{-}$ reductase, ${\mathrm{NO}}_{3}^{-}$-amended cultures produce N_2_O, representing up to 6% of the N-input. ${\mathrm{NO}}_{2}^{-}$ was shown to be the key intermediate of this production process. Upregulation in the expression of c*norB1*, *cnorB2, nnrS* and *norR* during the growth and the N_2_O accumulation phases suggests NO production in strain JAM1 cultures.

**Discussion:**

By showing that all the three denitrification reductases are active, this demonstrates that *M. nitratireducenticrescens* JAM1 is one of many bacteria species that maintain genes associated primarily with denitrification, but not necessarily related to the maintenance of the entire pathway. The reason to maintain such an incomplete pathway could be related to the specific role of strain JAM1 in the denitrifying biofilm of the denitrification reactor from which it originates. The production of N_2_O in strain JAM1 did not involve Nar, contrary to what was demonstrated in *Escherichia coli*. *M. nitratireducenticrescens* JAM1 is the only reported *Methylophaga* species that has the capacity to grow under anoxic conditions by using ${\mathrm{NO}}_{3}^{-}$ and N_2_O as sole electron acceptors for its growth. It is also one of a few marine methylotrophs that is studied at the physiological and genetic levels in relation to its capacity to perform denitrifying activities.

## Introduction

The complete denitrification pathway describes the successive reduction of nitrate (${\mathrm{NO}}_{3}^{-}$) to nitrite (${\mathrm{NO}}_{2}^{-}$), nitric oxide (NO), nitrous oxide (N_2_O), and nitrogen (N_2_) (Van Spanning, Delgado & Richardson, 2005). This process is used by bacteria for respiration in environments with low oxygen concentrations and with ${\mathrm{NO}}_{3}^{-}$ as an electron acceptor. The process is driven by metalloenzymes ${\mathrm{NO}}_{3}^{-}$ reductase, ${\mathrm{NO}}_{2}^{-}$ reductase, NO reductase, and N_2_O reductase (Einsle & Kroneck, 2004). As a facultative trait, denitrification occurs frequently across environments and is performed by bacteria of diverse origins (Zumft, 1997). However, numerous bacterial strains have been isolated with incomplete denitrification pathway, meaning that at least one reductase-encoding gene cluster is missing. As proposed by Zumft (Zumft, 1997), the four steps of reduction from ${\mathrm{NO}}_{3}^{-}$ to N_2_ could be seen as a modular assemblage of four partly independent respiratory processes that respond to combinations of different external and internal signals. This could explain the vast diversity of bacteria with incomplete denitrification pathway that can sustain growth with one of the four nitrogen oxides as electron acceptor. Another purpose of the incomplete pathway is related to detoxification, as ${\mathrm{NO}}_{2}^{-}$ and NO are deleterious molecules (Kaspar, 1982; Poole, 2005; Schreiber et al., 2012; Simon & Klotz, 2013).

*Methylophaga nitratireducenticrescens* JAM1 is a marine methylotrophic gammaproteobacterium that was isolated from a naturally occurring multispecies biofilm that has developed in a methanol-fed, fluidized denitrification system that treated recirculating water of the marine aquarium in the Montreal Biodome (Auclair et al., 2010; Villeneuve et al., 2013). This biofilm is composed of at least 15 bacterial species and of numerous protozoans (Labbé et al., 2003; Laurin et al., 2008), among which *Methylophaga* spp. and *Hyphomicrobium* spp. compose more than 50% of the biofilm (Labbé et al., 2007). Along with the denitrifying bacterium *Hyphomicrobium nitrativorans* NL23, *M. nitratireducenticrescens* JAM1 was shown to be the representative of the *Methylophaga* population in the biofilm (Auclair et al., 2010).

*M. nitratireducenticrescens* JAM1 is considered as a nitrate respirer as it can grow under anoxic conditions through the reduction of ${\mathrm{NO}}_{3}^{-}$ to ${\mathrm{NO}}_{2}^{-}$, which accumulates in the culture medium (Auclair et al., 2010). This trait is correlated with the presence of two gene clusters encoding dissimilatory nitrate reductases (*narGHJI*, referred as Nar1 and Nar2) in the genome of *M. nitratireducenticrescens* JAM1, which we showed that both contribute to ${\mathrm{NO}}_{3}^{-}$ reduction during strain JAM1 growth (Mauffrey, Martineau & Villemur, 2015). Anaerobic growth by strain JAM1 is a unique among *Methylophaga* spp. that were described as strictly aerobic bacteria (Boden, 2012). Genome annotation revealed that strain JAM1 seems to maintain an incomplete denitrification pathway with the presence of gene clusters encoding two putative cytochrome *bc*-type complex NO reductase (cNor) (*cnor1* and *cnor2*) and one putative dissimilatory N_2_O reductase, but lacks gene encoding a dissimilatory copper- (NirK) or cytochrome cd1-type (NirS) ${\mathrm{NO}}_{2}^{-}$ reductase. These gene clusters have been shown to be transcribed. However, the capacity of *M. nitratireducenticrescens* JAM1 to consume NO and N_2_O has not been fully determined. In addition to these gene clusters, genes involved in the NO response such as *nnrS* and *norR* are present (Mauffrey, Martineau & Villemur, 2015) suggesting tight regulation of denitrification genes such as *cnorB* and *nosZ*. Finally, the genome has a gene cluster encoding assimilatory nitrate and NADH-dependent nitrite reductases.

In this study, we assessed further the denitrification activities of strain JAM1 in pure cultures by demonstrating the consumption of NO and N_2_O by strain JAM1 in cultures amended with N_2_O or sodium nitroprusside as NO provider. Through our investigation, we found that strain JAM1 cultured with ${\mathrm{NO}}_{3}^{-}$ generates a small amount of N_2_O. We assessed whether nitrate and the nitrate reductases, nitrite and ammonium are directly involved in this N_2_O production, and found that ${\mathrm{NO}}_{2}^{-}$ is a key intermediate of this production process. Finally, we showed that the N_2_O accumulation/consumption cycle in ${\mathrm{NO}}_{3}^{-}$-amended cultures affects the expression of denitrification genes c*norB* (c*norB1* and *cnorB2*) and *nosZ,* and also *nnrS* and *norR*, which encode NO-sensitive regulators. These results suggest that NO is also generated in ${\mathrm{NO}}_{3}^{-}$-amended cultures.

## Materials and Methods

### Bacterial growth conditions

*M. nitratireducenticrescens* JAM1 and the JAM1Δ*narG1narG2* double mutant were cultured in the American Type Culture Collection (ATCC, Manassas, VA, USA) *Methylophaga* medium 1403 (Villeneuve et al., 2013; Mauffrey, Martineau & Villemur, 2015). When required, ${\mathrm{NO}}_{3}^{-}$ (NaNO_3_) or ${\mathrm{NO}}_{2}^{-}$ (NaNO_2_) (Fisher Scientific Canada, Ottawa, ON, Canada) were added to the medium. Medium (40 or 60 mL) was dispensed into 720-mL bottles (680- or 660-mL head space) that were sealed with caps equipped with septum and which were then autoclaved. After autoclaving, the following filter-sterilized solutions were added to the bottles (40 mL volume): 120 µL methanol (final concentration 0.3% [vol/vol]; 74.3 mM), 800 µL solution T (per 100 mL: 0.7 g KH_2_PO_4_, 10 g NH_4_Cl, 10 g Bis-Tris, 0.3 g ferric ammonium citrate (pH 8)), 400 µL Wolf’s mineral solution (pH 8) (ATCC), and 40 µL vitamin B_12_ (stock solution 0.1 mg/mL). The Wolf mineral solution is composed of (per liter) 0.5 g EDTA, 3.0 g MgSO_4_.7H_2_O, 0.5 g MnSO_4_.H_2_O, 1.0 g NaCl, 0.1 g FeSO_4_.7H_2_O, 0.1 g Co(NO_3_)_2_.6H2O, 0.1 g CaCl_2_ (anhydrous), 0.1 g ZnSO_4_.7H_2_O, 0.010 g CuSO_4_.5H_2_O, 0.010 g AlK(SO_4_)_2_ (anhydrous), 0.010 g H_3_BO_3_, 0.010 g Na_2_MoO_4_.2H2O, 0.001 g Na_2_SeO_3_ (anhydrous), 0.010 g Na_2_WO_4_.2H_2_O, and 0.020 g NiCl_2_.6H_2_O. The final concentration of ammonium (${\mathrm{NH}}_{4}^{+}$) in the *Methylophaga* 1403 medium was measured as 21 mg-N vial^−1^ (20.9 mg-N vial^−1^ from NH_4_Cl and 0.1 mg-N vial^−1^ from ferric ammonium citrate). The amount of NO${}_{3}^{-}$ carried by the Wolf mineral solution (0.0038 mg-N vial^−1^) was deemed negligible. For the anoxic cultures, bottles were flushed with nitrogen gas (N_2_, purity >99.9%; Praxair, Mississauga, ON, Canada) or argon (purity 99.9%, Praxair) for 20 min prior to autoclaving. When necessary, N_2_O (purity 99.9%, Praxair) and acetylene (10% [vol/vol] of headspace; Praxair) were injected into the headspace before autoclaving. Acetylene is an inhibitor of nitrous oxide reductase and has been extensively used in N_2_O studies to observe N_2_O production in cells (Klemedtsson et al., 1977). Inoculums were made from fresh culture cultivated under oxic conditions without ${\mathrm{NO}}_{3}^{-}$ to reach an optical density (OD_600_) of 0.025. Culture bottles were incubated at 30 °C in the dark. For oxic cultures, bottles were shaken at 150 rpm.

The capacity for strain JAM1 to reduce NO was tested with sodium nitroprusside (sodium nitroprusside hypochloride ([SNP]; purity ≥99.0%, Sigma-Aldrich, St. Louis, MO, USA) as the NO source. To avoid SNP toxicity, strain JAM1 was first cultured in *Methylophaga* 1403 medium under oxic conditions without ${\mathrm{NO}}_{3}^{-}$ for 24 h. The cells were then centrifuged (8,000 g 5 min) and dispersed into fresh medium supplemented with 2 mM, 5 mM, or no SNP. Culture medium with 5 mM SNP and no biomass was also used as a control. Cells were incubated under oxic conditions at 30 °C in the dark, and N_2_O production was monitored. To investigate the potential role of ${\mathrm{NH}}_{4}^{+}$ in N_2_O production, NH_4_Cl-free cultures were employed under oxic and anoxic conditions using solution T containing no NH_4_Cl. Prior to inoculation, cells from start-up cultures were centrifuged and rinsed three times with saline solution to remove any residual traces of ${\mathrm{NH}}_{4}^{+}$.

Bacterial growth was monitored by spectrophotometry (OD_600_). Bacterial flocs were dispersed with a Potter-Elvehjem homogenizer prior to measurement. Oxygen concentrations in the headspace were monitored in cultures under oxic conditions by gas chromatography using a temperature conductivity detector (7890B series GC Custom, SP1 option 7890-0504/0537; Agilent Technologies, Mississauga, ON, Canada). Although vials were capped in the oxic cultures, O_2_ concentrations in the headspace (680 ml) did not significantly decrease (T0 *h* = 20.4 ± 0.3%; T100 *h* = 19.7 ± 0.9%).

### ^15^N-labeling of N_2_O

Strain JAM1 cultures were made with 22 mg-N vial^−1^ Na^15^NO_3_ (Sigma-Aldrich) in NH_4_Cl-free medium or with 22 mg-N vial^−1^ Na^14^NO_3_ and 20.7 mg-N vial^−1^ ^15^NH_4_Cl (Sigma-Aldrich). Both cultures were used under anoxic conditions, and 10% (vol/vol) acetylene was added to allow N_2_O to accumulate. Cultures were made in triplicate. After 14 days of incubation, the headspace of each replicate was pooled, and 100 mL of the gaseous phase was sampled in Tedlar bags. N_2_O-isotope measurements were performed at the Environmental Isotope Laboratory (Earth & Environmental Sciences; University of Waterloo, ON, Canada) via Trace Gas-GVI IsoPrime-Isotope Ratio Mass Spectrometry (TG-IRMS). ^45^[N_2_O]/^44^[N_2_O] and ^46^[N_2_O]/^44^[N_2_O] ratios were calculated according to the peak intensity measured for ^46^[N_2_O], ^45^[N_2_O] and ^44^[N_2_O]. The ^15^N/^14^N isotopic ratio was derived from the previous results from Eq. (1).

(1) \begin{eqnarray*}Rs=\sum ({\text{}}^{15}\mathrm{N} {\mathrm{vial}}^{-1})/\sum ({\text{}}^{14}{\text{N vial}}^{-1})=[{\text{}}^{15}\mathrm{N}45+2({\text{}}^{15}\mathrm{N}46)]/[2({\text{}}^{14}\mathrm{N}44)+{}^{14}\mathrm{N}45]\end{eqnarray*}

where Rs is the sample isotopic ratio. Calculated from the ^45^[N_2_O]/^44^[N_2_O] and ^46^[N_2_O]/^44^[N_2_O] isotopic ratios, ^14^N45 is the quantity of ^14^N in ^45^[N_2_O], ^15^N45 is the quantity of ^15^N in ^45^[N_2_O], ^14^N44 is the quantity of ^14^N in ^44^[N_2_O] and ^15^N46 is the quantity of ^15^N in ^46^[N_2_O]. We considered the isotope fractionation by denitrification enzymes as negligible in our calculations (delta values ranging from −10‰ to −40‰) (Snider, Schiff & Spoelstra, 2009).

### Measurements of nitrogenous compounds

${\mathrm{NO}}_{3}^{-}$ and ${\mathrm{NO}}_{2}^{-}$ concentrations were determined by ion chromatography using the 850 Professional IC (Metrohm, Herisau, Switzerland) with a Metrosep A Supp 5 analytical column (250 mm × 4.0 mm).

N_2_O and N_2_ concentrations were determined by gas chromatography. Headspace samples (10 mL) were collected using a Pressure Lok gastight glass syringe (VICI Precision Sampling Inc., Baton Rouge, LA, USA) and were injected through the injection port of a gas chromatograph equipped with a thermal conductivity detector and electron-capture detector (7890B series GC Custom, SP1 option 7890-0504/0537; Agilent Technologies). The reproducibility of the N_2_O was assessed before each set of measurements via the repeated analysis of certified N_2_O standard gas with standard deviations <5%. N_2_O standards (500 ppmv and 250 ppmv) were created based on dilutions from the 10,000 ppmv N_2_O stock standard. The 10,000 ppmv stock standard was obtained by injecting 1% pure N_2_O (Praxair) into a 720 mL gastight bottle. The detection limit of the N_2_O was set to <10 ppbv, corresponding to the 0.3 nmol/vial composition of our bioassays. No significant N_2_O production patterns were observed through our blank experiments involving sterile media and empty glass bottles. The total quantity of N_2_O in the culture bottle (aqueous phase and headspace) (X_N2O_ in µmole vial^−1^) was calculated according to Eq. (2).

(2) \begin{eqnarray*}{\mathrm{X}}_{\mathrm{N2O}}=[{\mathrm{K}}_{\mathrm{cpH30sw}}\ast {\mathrm{A}}_{\mathrm{N2O}}\ast \mathrm{P}\ast {\mathrm{V }}_{1}]_{\mathrm{aq}}+[{A}_{\mathrm{N2O}}\ast {\mathrm{V }}_{\mathrm{g}}/{\mathrm{V }}_{\mathrm{n}}]_{\mathrm{gaz}}\end{eqnarray*}

where A_N2O_: the N_2_O mixing ratio measured in the headspace (µmole_N2O_ mole^−1^); P: 1 atm; V_1_ and V_g_: volume of the aqueous (0.04 or 0.06 L vial^−1^) and gaseous phases (0.68 or 0.66 L vial^−1^), respectively; and V_n_: molar volume (RT (gas constant): 0.08206 L atm K^−1^ mol^−1^ * 303 K = 24.864 L mol^−1^). K_H30sw_ is the corrected Henry’s constant for seawater at 30 °C (0.01809 mol L^−1^ atm^−1^) according to Weiss and Price (1980). X_N2O_ was then converted (Eq. (3)) into mg-N vial^−1^ for an easier calculation of mass balances using the other nitrogenous compounds:

(3) \begin{eqnarray*}{\mathrm{X}}_{\mathrm{N}-\mathrm{N2O}}={X}_{\mathrm{N2O}}\ast [2\mathrm{N}/{\mathrm{N}}_{2}\mathrm{O}]\ast [0.014 \mathrm{mg}-\mathrm{N} \mathrm{\mu }{\mathrm{mole}}^{-1}].\end{eqnarray*}

The reproducibility of the N_2_ was assessed before each set of measurements via a repeated analysis of N_2_ (purity >99.99%, Praxair) diluted in a 720 mL gastight bottle (0 and 500 ppmv) flushed with argon (purity >99.99%, Praxair). The total quantity of N_2_ in the culture bottles was only considered for the headspace, as the quantity of dissolved N_2_ in the aqueous phase was considered to be negligible in our experimental design based on Henry’s constant (0.0005 mol L^−1^ atm^−1^) and was thus calculated according to Eq. (4).

(4) \begin{eqnarray*}{\mathrm{X}}_{\mathrm{N}\text{-}\mathrm{N2}}(\mathrm{mg}\text{-}\mathrm{N} {\mathrm{vial}}^{-1})=[{\mathrm{A}}_{\mathrm{ N2}}\ast {\mathrm{V }}_{\mathrm{g}}/{\mathrm{V }}_{\mathrm{n}}]_{\mathrm{gaz}}\ast [2\mathrm{N}/{\mathrm{N}}_{2}]\ast [0.014 \mathrm{mg}-\mathrm{N} \mathrm{\mu }{\mathrm{mole}}^{-1}].\end{eqnarray*}

### RNA extraction

Anoxic cultures of strain JAM1 were created in an NH_4_Cl-free 1403 medium supplemented with 22 mg-N vial^−1^ ${\mathrm{NO}}_{3}^{-}$. Cells were harvested at specific times, and RNA was immediately extracted using the PureLink RNA mini kit (Ambion Thermo Fisher Scientific, Burlington, ON, Canada). RNA extracts were treated twice with TurboDNase (Ambion), and RNA quality was verified by agarose gel electrophoresis. The absence of remaining DNA was checked via the end-point polymerase chain reaction (PCR) amplification of the 16S rRNA gene using RNA extracts as the template.

### Gene expression

cDNAs samples were generated from the RNA using hexameric primers and the Reverse Transcription System developed by Promega (Madison, WI, USA) with 1 µg of RNA and quantified by spectrophotometry. Real-time quantitative PCR (qPCR) assays were performed using the Faststart SYBR Green Master (Roche Diagnostics, Laval, QC, Canada) according to the manufacturer’s instructions. All reactions were performed in a Rotor-Gene 6000 real-time PCR thermocycler (Qiagen Inc. Toronto, ON, Canada), and each reaction contained 25 ng of cDNA and 300 nM of primers (Table 1). Genes tested included c*norB1*, c*norB2*, *nnrS*, *nosZ, norR* and *nr*, and the reference genes *dnaG*, *rpoD* and *rpoB* (Mauffrey, Martineau & Villemur, 2015) and the PCR began with an initial denaturation step of 10 min at 95 °C followed by 40 cycles of 10 s at 95 °C, 15 s at 60 °C, and 20 s at 72 °C. To confirm the purity of the amplified products, a melting curve analysis was performed by increasing the temperature from 65 °C to 95 °C at increments of 1 °C per step with a pause of 5 s included between each step. All genes for each sample and standard were tested in a single run. The amplification efficiency level was tested for each set of primer pairs by qPCR using a dilution of strain JAM1 genomic DNA as the template. The amplification efficiencies for all primer pairs varied between 0.9 and 1.1. The copy number of each gene was calculated according to standard curves using dilutions of strain JAM1 genomic DNA. The gene expression levels of the targeted genes were standardized with the three reference genes. The RNA extraction and qPCR were performed with three to four independent biological replicates. The significance of differential expression levels was tested for each phase against the pre-culture phase using One-way ANOVA tests with Tukey *post hoc* tests.

**Table 1:** Primers used for RT-qPCR.

Primers	Target gene	Locus tag^*^	Sequence (5′–3′)
cytochrome *bc*-type complex NO reductases			
cnorB1-510f	c*norB1*	Q7A_0433	CCTGATCGGTTTGGCTCTC
cnorB1-635r			CCCATGATCAATTCCCAGAC
cnorB2-334f	c*norB2*	Q7A_0487	GGCAACAAGCTATTGGAGCA
cnorB2-449r			GTGGTGGTAAAGCGACCAGA
N_2_O reductase			
nosZ-826f	*nosZ*	Q7A_0459	GAGCGTGACTGGGTAGTCGT
nosZ-952r			GTGTCAACTCGCTCCCTTTG
NO-sensitive regulators			
nnrs-749f	*nnrS*	Q7A_1801	TGTTCGCCATTTCAGCAATA
nnrs-848r			TAACCGATGTGCAAAGACCA
norR-265f	*norR*	Q7A_0435	CGGTTTGCTGCAGATAGTGA
norR-386r			CCCCAGGGCCTGTTATTTAT
Assimilatory nitrate reductase			
nr-1350f	*nr*	Q7A_2619	ATTCGGTACAGTCGGTTTGC
nr-1474r			TGTCTGGATTATTGCCACCA
Reference genes			
dnaG-774f	*dnaG*	Q7A_342	CATCCTGATCGTGGAAGGTT
dnaG-894r			GCTGCGAATCAACTGACGTA
rpob (3861F)	*rpoB*	Q7A_2329	TGAGATGGAGGTTTGGGCAC
rpob (4006R)			GCATACCTGCATCCATCCGA
rpoD (10F)	*rpoD*	Q7A_343	CAGCAATCACGCGTTAAAGA
rpoD(153R)			ACCCAGGTCGCTGAACATAC

**Notes.**

* from GenBank accession number CP003390.3.

## Results

### *M. nitratireducenticrescens* JAM1 grows on N_2_O under anoxic conditions

Strain JAM1 was cultured under anoxic conditions with either ${\mathrm{NO}}_{3}^{-}$ in the medium or with N_2_O injected in the headspace as the sole electron acceptor. Both types of culture received the same electron equivalent of ${\mathrm{NO}}_{3}^{-}$ or N_2_O (1.3 mmole vial^−1^ or 18.2 and 36.4 mg-N vial^−1^, respectively) according to:

(5) \begin{eqnarray*}& & {\mathrm{NO}}_{3}^{-}+2{\mathrm{e}}^{-}+2{\mathrm{H}}^{+}\rightarrow {\mathrm{NO}}_{2}^{-}+{\mathrm{H}}_{2}\mathrm{O}\end{eqnarray*}

(6) \begin{eqnarray*}& & {\mathrm{N}}_{2}\mathrm{O}+2{\mathrm{e}}^{-}+2{\mathrm{H}}^{+}\rightarrow {\mathrm{N}}_{2}+{\mathrm{H}}_{2}\mathrm{O}.\end{eqnarray*}

**Figure 1:**
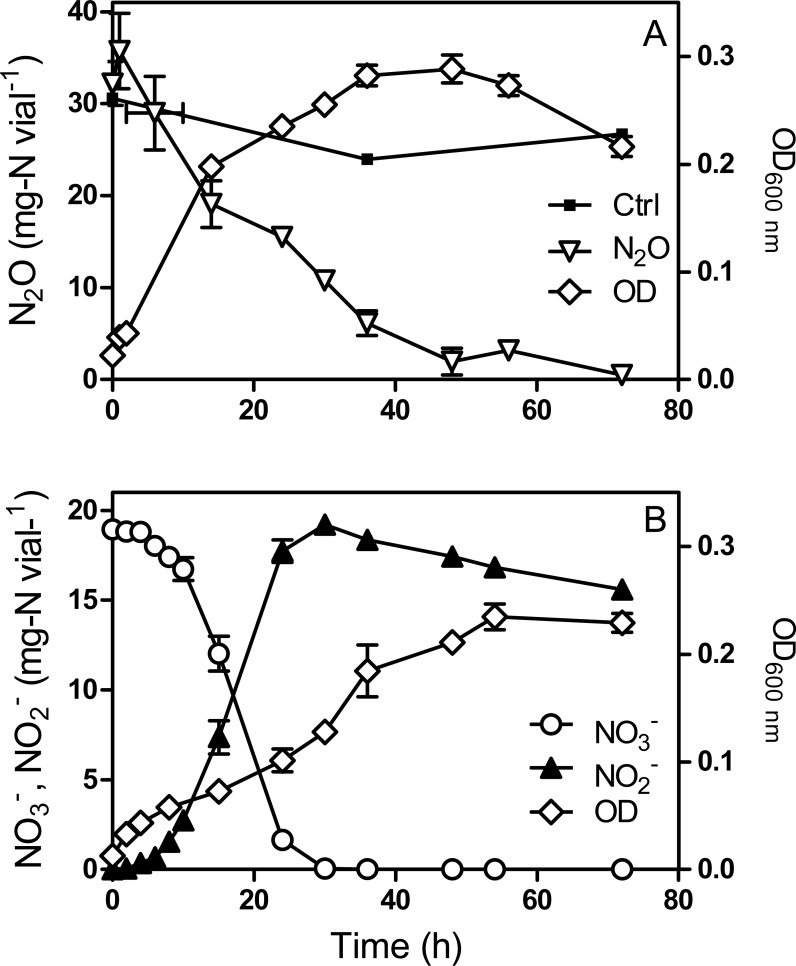
*Methylophaga nitratireducenticrescens* JAM1 growth with N_2_O or ${\mathrm{NO}}_{3}^{-}$ as an electron acceptor. Strain JAM1 was cultured with 36.4 mg-N vial^−1^ N_2_O (A) or 18.2 mg-N vial^−1^ ${\mathrm{NO}}_{3}^{-}$ (B) under anoxic conditions. N_2_O, ${\mathrm{NO}}_{3}^{-}$ and ${\mathrm{NO}}_{2}^{-}$ concentrations and growth were measured over different time intervals. Control (A): N_2_O injected in non-inoculated vials. To minimize oxygen contamination, sampling was performed using a glove bag inflated with nitrogen gas. Data represent mean values ± standard deviation (SD; *n* = 3).

In N_2_O-amended cultures, N_2_O decrease was apparent from the start and consumption continued for 48 h (Fig. 1A). The N_2_O decrease paralleled strain JAM1 growth with almost complete N_2_O consumption. The ${\mathrm{NO}}_{3}^{-}$-amended cultures showed complete ${\mathrm{NO}}_{3}^{-}$ consumption and equivalent ${\mathrm{NO}}_{2}^{-}$ accumulation after 24 h (Fig. 1B). However, slower growth than that recorded for the N_2_O cultures was observed. Such growth kinetics could be related to the toxicity of ${\mathrm{NO}}_{2}^{-}$ that accumulated in the medium. Both types of culture reached equivalent biomass concentration (*t* test on the last 4-time points, *P* > 0.05).

### *M. nitratireducenticrescens* JAM1 consumes N_2_O under oxic conditions

**Figure 2:**
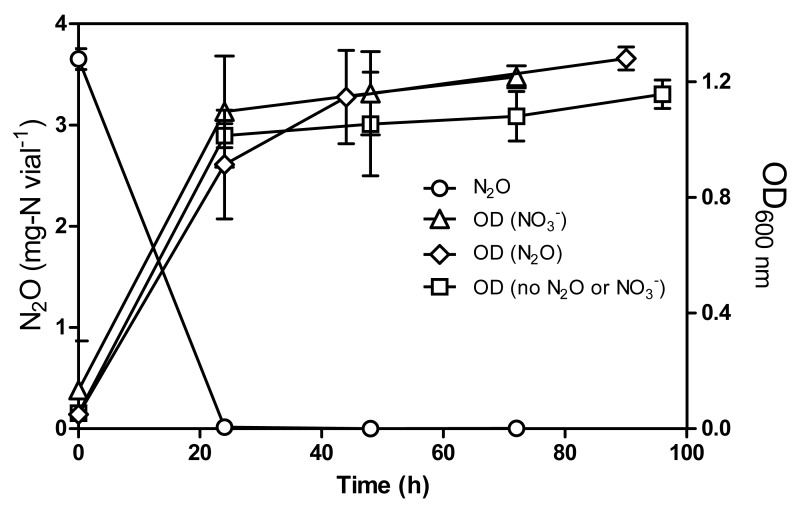
N_2_O consumption by *Methylophaga nitratireducenticrescens* JAM1 under oxic conditions. Strain JAM1 was cultured with 3.5 mg-N vial^−1^ N_2_O, with 22 mg-N vial^−1^ ${\mathrm{NO}}_{3}^{-}$ or without N_2_O and ${\mathrm{NO}}_{3}^{-}$, and under oxic conditions. N_2_O concentration was measured in N_2_O-amended cultures only. Growth were measured over different time intervals. Data represent mean values ± SD (*n* = 3).

In a previous study, Mauffrey, Martineau & Villemur (2015) demonstrated that strain JAM1 can consume ${\mathrm{NO}}_{3}^{-}$ under oxic growth conditions with equivalent accumulation of ${\mathrm{NO}}_{2}^{-}$. We tested if this was also the case with N_2_O. Culturing strain JAM1 under oxic conditions with N_2_O (3.5 mg-N vial^−1^) showed a complete N_2_O consumption within 24 h (Fig. 2). Growth patterns illustrated in Fig. 2 were similar between oxic cultures amended with or without either N_2_O or ${\mathrm{NO}}_{3}^{-}$. In the presence of O_2_, cultures reached higher (4–5 times) biomass concentration than the anoxic cultures.

### N_2_O production in ${\mathrm{NO}}_{3}^{-}$-amended cultures

**Figure 3:**
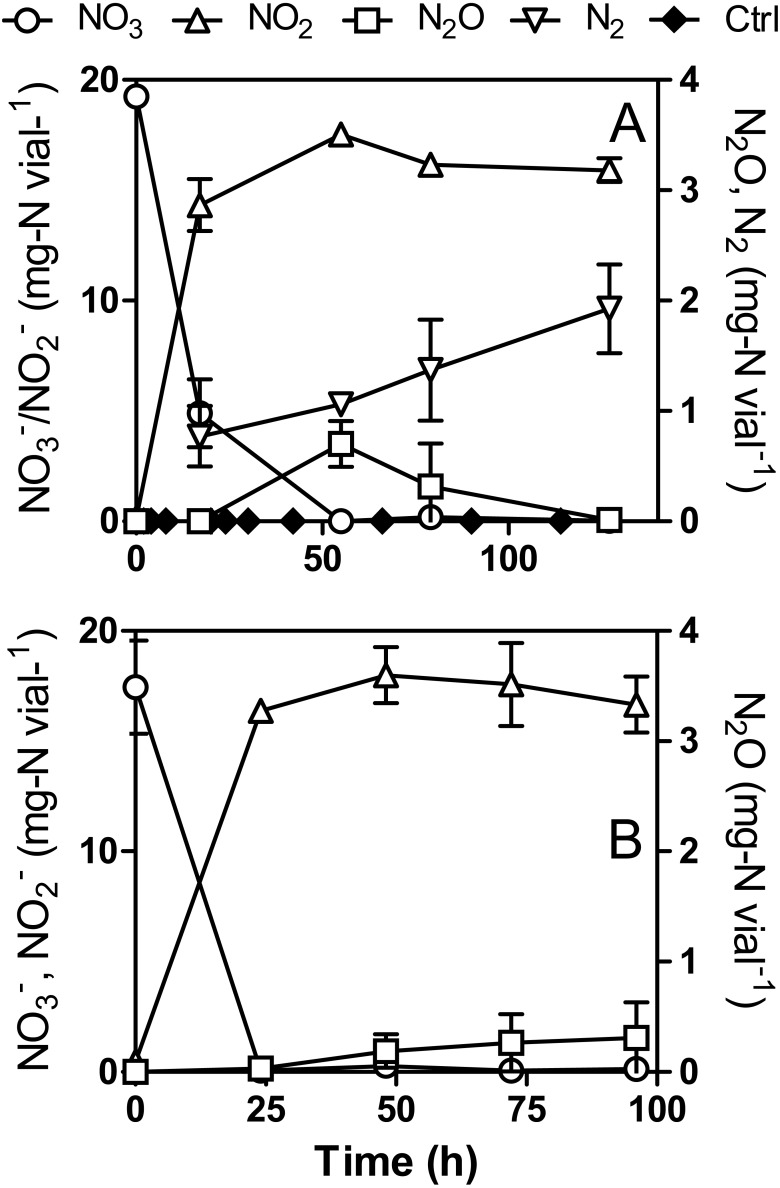
N_2_O production by *Methylophaga nitratireducenticrescens* JAM1. Strain JAM1 was cultured under anoxic (A) or oxic (B) conditions with ${\mathrm{NO}}_{3}^{-}$ (22 mg-N vial^−1^). ${\mathrm{NO}}_{3}^{-}$, ${\mathrm{NO}}_{2}^{-}$ and N_2_O concentrations were measured over different time intervals. N_2_ concentration was measured in anoxic cultures that were flushed with argon. Ctrl: Abiotic control with 18.2 mg-N vial^−1^ ${\mathrm{NO}}_{2}^{-}$. Data represent mean values ± SD (*n* = 3).

During the first assays to test the capacity of strain JAM1 to reduce N_2_O under anoxic conditions, cultures were performed with N_2_O (3.5 mg-N vial^−1^) but with the addition of ${\mathrm{NO}}_{3}^{-}$ (20 mg-N vial^−1^) to make sure that growth would occur. Although N_2_O was completely consumed within 24 h, a net production of N_2_O was observed after 48 h. To further investigate this observation, strain JAM1 was cultured under anoxic conditions with ${\mathrm{NO}}_{3}^{-}$, and ${\mathrm{NO}}_{3}^{-}$, ${\mathrm{NO}}_{2}^{-}$ and N_2_O were measured (Fig. 3A). Complete ${\mathrm{NO}}_{3}^{-}$ reduction (19.3 ± 0.3 mg-N vial^−1^) was performed within 55 h. The ${\mathrm{NO}}_{2}^{-}$ level reached 17.5 ± 0.2 mg-N vial^−1^ over this period and decreased slowly to 15.9 ± 0.5 mg-N vial^−1^. N_2_O production initiated when ${\mathrm{NO}}_{3}^{-}$ was nearly reduced and reached 0.70 ± 0.21 mg-N vial^−1^ after 55 h of incubation (Fig. 3A). N_2_O was completely reduced after 127 h. In parallel, for cultures in which the headspace was flushed with argon, N_2_ production was also measured. The corresponding results show an increase of N_2_ in the headspace (Fig. 3A) by 1.14 ± 0.54 mg-N vial^−1^ after 127 h, which represent 6.0 ± 2.9% of the N input. As the *Methylophaga* 1403 medium contains ferrous chloride (216 µmole vial^−1^), N_2_O production could originate from the abiotic reaction between ${\mathrm{NO}}_{2}^{-}$ that accumulated in the cultures and the ferrous ion (Klueglein et al., 2014). An abiotic control was performed with 18.2 mg-N vial^−1^ ${\mathrm{NO}}_{2}^{-}$. N_2_O was detected in the abiotic control after 20 h and reached 0.00172 ± 0.00012 mg-N vial^−1^ after 114 h, which is 407 times lower than the N_2_O concentration measured in the anoxic cultures. This results showed that the abiotic reaction generated negligible amount of N_2_O.

Under oxic conditions, ${\mathrm{NO}}_{3}^{-}$ reduction (17.4 ± 2.1 mg-N vial^−1^) was complete after 24 h with equivalent ${\mathrm{NO}}_{2}^{-}$ accumulation (17.1 ± 1.3 mg-N vial^−1^). N_2_O production started after complete ${\mathrm{NO}}_{3}^{-}$ reduction (Fig. 3B) and increased to reach 0.31 ± 0.32 mg-N vial^−1^ after 96 h of incubation (1.7% of N input). Unlike trends observed for the anoxic cultures, N_2_O concentration did not decrease in the oxic cultures. N_2_O production and consumption could have reached an equilibrium and loss of nitrogen would occur by N_2_ production.

### ${\mathrm{NO}}_{3}^{-}$, ${\mathrm{NO}}_{2}^{-}$ and N_2_O dynamics in NH_4_Cl-free cultures

**Figure 4:**
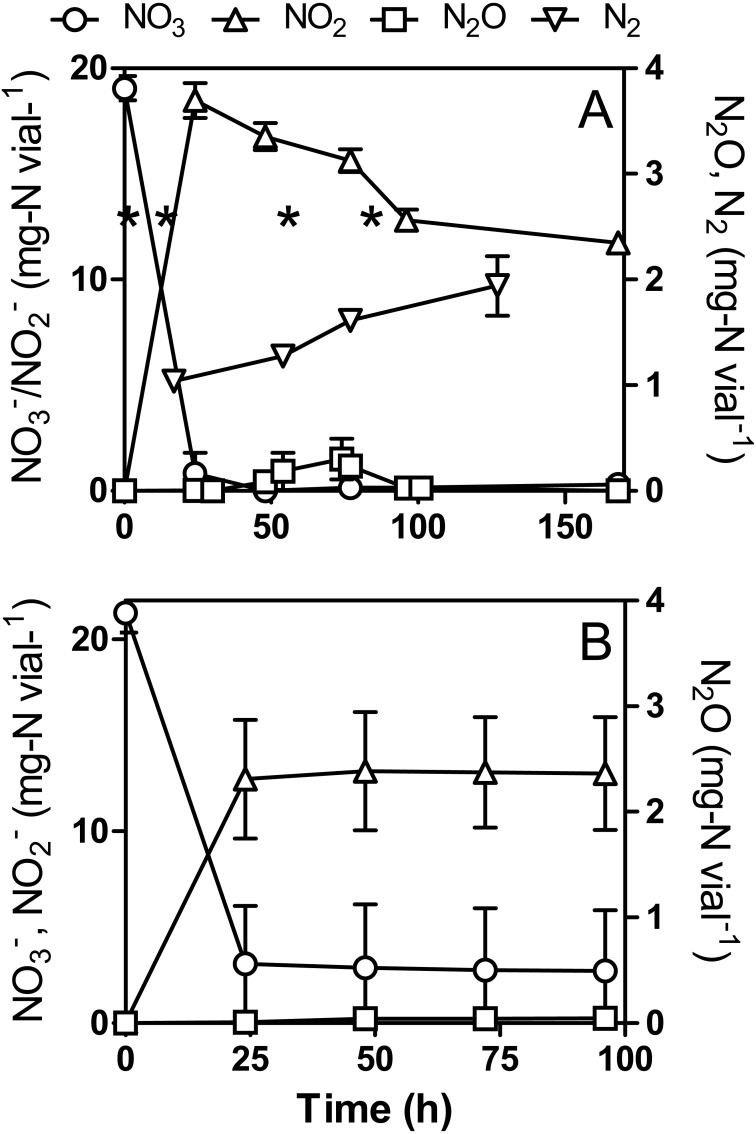
${\mathrm{NO}}_{3}^{-}$, ${\mathrm{NO}}_{2}^{-}$ and N_2_O dynamics by *Methylophaga nitratireducenticrescens* JAM1 in NH_4_Cl-free cultures. Strain JAM1 was cultured under anoxic (A) or oxic (B) conditions with ${\mathrm{NO}}_{3}^{-}$ (22 mg-N vial^−1^) in NH_4_Cl-free 1403 medium. ${\mathrm{NO}}_{3}^{-}$, ${\mathrm{NO}}_{2}^{-}$ and N_2_O concentrations were measured over different time intervals. N_2_ concentration was measured in anoxic cultures that were flushed with argon. The results are derived from triplicate cultures. In (A) asterisks denote the sampling times used for RNA extraction (see Fig. 6). Data represent mean values ± SD (*n* = 3).

The original 1403 medium recommended by the ATCC for culturing *Methylophaga* spp. contains 20.9 mg-N vial^−1^ NH_4_Cl and 0.1 mg-N vial^−1^ ferric ammonium citrate (see ‘Material and Methods’). Based on the deduced nitrogen metabolic pathways from strain JAM1 genome (Fig. S1), N-assimilation into the biomass should proceed directly from ${\mathrm{NH}}_{4}^{+}$ and minimal ${\mathrm{NO}}_{3}^{-}$ reduction to ${\mathrm{NH}}_{4}^{+}$ would be occurring. For the next set of experiments, we aimed to determine the effect of removing NH_4_Cl, which provides most of the ${\mathrm{NH}}_{4}^{+}$ (99.5%), on the dynamics of ${\mathrm{NO}}_{3}^{-}$, ${\mathrm{NO}}_{2}^{-}$ and N_2_O. We hypothesized that forcing strain JAM1 to reroute some ${\mathrm{NO}}_{3}^{-}$ for N assimilation would affect denitrification and thus growth rates. Strain JAM1 was cultured with *ca.* 20 mg-N vial^−1^ ${\mathrm{NO}}_{3}^{-}$ under anoxic or oxic conditions in NH_4_Cl-free medium (Fig. 4). Growth pattern observed under anoxic conditions was similar between the regular and NH_4_Cl-free cultures, as also the growth pattern under oxic conditions between the regular and NH_4_Cl-free cultures.

Under anoxic NH_4_Cl-free conditions, full ${\mathrm{NO}}_{3}^{-}$ reduction (19.1 ± 0.6 mg-N vial^−1^) occurred within 48 h (Fig. 4A). The N_2_O profile found was similar to that observed in regular cultures (Fig. 3A), though lower N_2_O concentrations were detected during the accumulating phase. The ${\mathrm{NO}}_{2}^{-}$ level reached 18.5 ± 0.8 mg-N vial^−1^ after 24 h and then slowly decreased to 12.8 ± 0.5 mg-N vial^−1^ after 96 h. Cultures flushed with argon showed an increase of N_2_ in the headspace (Fig. 4A) by 0.90 ± 0.28 mg-N vial^−1^ after 127 h, which is similar to N_2_ production in the regular culture medium. Nitrogen assimilation by the biomass could account for the difference in nitrogen mass balance (28.3%).

Unlike the cultures in regular medium (Fig. 3B), NO${}_{3}^{-}$ (21.3 ± 1.0 mg-N vial^−1^) was not completely reduced under oxic NH_4_Cl-free conditions, and it stopped after 24 h at 2.9 ± 2.7 mg-N vial^−1^ (Fig. 4B). In conjunction with ${\mathrm{NO}}_{3}^{-}$ reduction, ${\mathrm{NO}}_{2}^{-}$ levels stopped accumulating at 13.0 ± 2.6 mg-N vial^−1^ after 24 h. N_2_O was observed after 48 h of incubation (Fig. 4B), after which it slowly accumulated and reached a concentration of 0.043 ± 0.048 mg-N vial^−1^. This level is seven times lower than that of the regular culture medium (Fig. 3B).

To assess whether N_2_O could have been generated through ${\mathrm{NH}}_{4}^{+}$, strain JAM1 was cultured under anoxic conditions with 22 mg-N vial^−1^ ${\mathrm{NO}}_{3}^{-}$, 20.7 mg-N vial^−1^ ^15^${\mathrm{NH}}_{4}^{+}$, and acetylene to prevent the reduction of N_2_O to N_2_. If ${\mathrm{NH}}_{4}^{+}$ is involved in N_2_O production, high proportion of labelled N_2_O is expected. If ${\mathrm{NH}}_{4}^{+}$ is not involved in N_2_O production, we expected the production of labeled N_2_O to be derived from ^15^${\mathrm{NO}}_{3}^{-}$ naturally present in Na${\mathrm{NO}}_{3}^{-}$ at a natural ^15^N/^14^N isotopic ratio of 0.0036765. In the ^15^${\mathrm{NH}}_{4}^{+}$-amended cultures, the ^45^[N_2_O]/^44^[N_2_O] and ^46^[N_2_O]/^44^[N_2_O] ratios measured were 0.008 and 0.0165, respectively, with an ^15^N/^14^N isotopic ratio of 0.020418. As a control, strain JAM1 cultured under anoxic conditions with ^15^${\mathrm{NO}}_{3}^{-}$ in NH_4_Cl-free medium with acetylene showed, as was expected, all N_2_O recovered in ^46^[N_2_O]. Because low ^15^N/^14^N isotopic ratio were found in the ^15^${\mathrm{NH}}_{4}^{+}$-amended cultures, our results suggest that N_2_O do not proceed through ${\mathrm{NH}}_{4}^{+}$.

### NO reduction by *M. nitratireducenticrescens* JAM1

**Figure 5:**
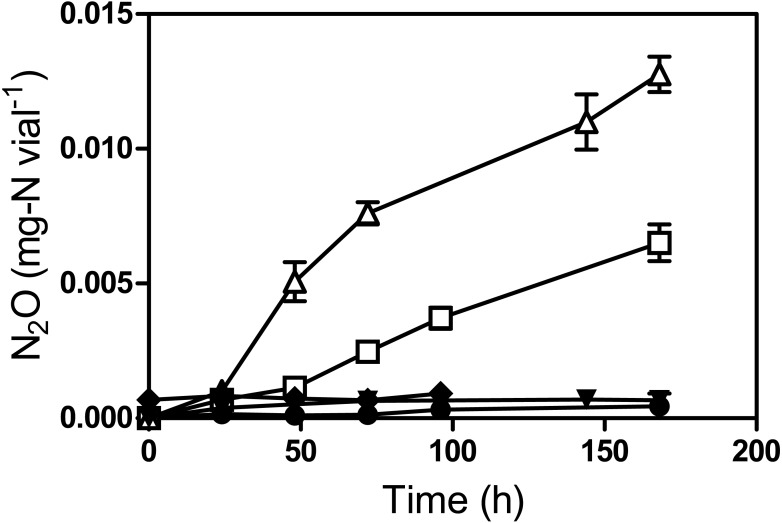
Reduction of NO to N_2_O by *Methylophaga nitratireducenticrescens* JAM1. Strain JAM1 was cultured under oxic conditions without ${\mathrm{NO}}_{3}^{-}$ and with 2 mM (square), with 5 mM (triangle), or with no (circle) sodium nitroprusside (SNP). N_2_O concentrations were measured over different time intervals. Controls with 5 mM SNP in non-inoculated culture medium (reverse triangle) and in culture medium inoculated with autoclaved biomass (diamond) were also performed. Data represent mean values ± SD (*n* = 3).

To verify NO reduction by strain JAM1, N_2_O generation was monitored in cultures without ${\mathrm{NO}}_{3}^{-}$ and supplemented with sodium nitroprusside hypochloride (SNP) used as an NO donor (Moore et al., 2004). Because N_2_O is quickly reduced under anoxic conditions but accumulates under oxic conditions, these assays were performed under oxic conditions (Fig. 5). N_2_O started to accumulate in both 2 mM and 5 mM SNP-supplemented media after 24 h of incubation, reaching 7.9 ± 0.5 µg-N vial^−1^ and 14.5 ± 0.4 µg-N vial^−1^, respectively, after 168 h. No N_2_O production was observed in strain JAM1 cultures without SNP or in the controls with non-inoculated culture medium supplemented with SNP or inoculated with autoclaved biomass.

**Figure 6:**
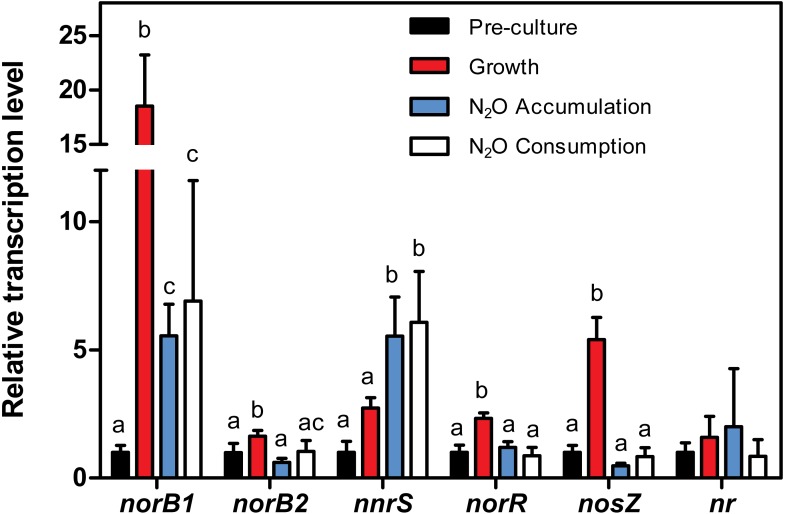
Relative transcript levels of c*norB1*, c*norB2*, *nnrS*, *nosZ*, *norR* and the assimilatory nitrate reductase (*nr*). Strain JAM1 was cultured under anoxic conditions in NH_4_Cl-free 1403 medium with 22 mg-N vial${}^{-1}{\mathrm{NO}}_{3}^{-}$. Growth patterns were similar to those shown in Fig. 1B under the same conditions with regular 1403 medium. Samples were drawn from the pre-cultures (2–3 day old oxic cultures with no ${\mathrm{NO}}_{3}^{-}$) and during the growth phase (${\mathrm{NO}}_{3}^{-}$ reduction), N_2_O accumulation phase, and N_2_O consumption phase (see Fig. 4A), from which total RNA was extracted for RT-qPCR assays. Changes in the levels of c*norB1*, c*norB2*, *nnrS*, *nosZ, norR* and *nr* transcripts were calculated relative to their expression during the pre-culture phase (set to one, black column). One-way ANOVA tests with Tukey *post hoc* tests were performed within each phase. Columns represented by different letters are significantly different (*P* < 0.05). Data represent mean values ± SD.

### Role of Nar systems in NO/N_2_O production

In the absence of NirK or NirS, N_2_O could have been generated via NO by the Nar system (see ‘Discussion’). We used the JAM1Δ*narG1narG2* double mutant, which lacks functional Nar-type nitrate reductases and which cannot grow under anoxic conditions (Mauffrey, Martineau & Villemur, 2015). Strain JAM1 and the JAM1Δ*narG1narG2* were cultured with 16.8 mg-N vial^−1^ ${\mathrm{NO}}_{3}^{-}$ under oxic conditions. The growth of strain JAM1 and the mutant was similar (Mauffrey, Martineau & Villemur, 2015). After 96 h of incubation, strain JAM1 completely reduced ${\mathrm{NO}}_{3}^{-}$ to ${\mathrm{NO}}_{2}^{-}$ and produced 0.14 mg-N vial^−1^ of N_2_O (Table 2). As was expected, ${\mathrm{NO}}_{3}^{-}$ was not reduced, and ${\mathrm{NO}}_{2}^{-}$ was not produced by JAM1Δ*narG1narG2*. Contrary to the wild type strain, the mutant did not produce N_2_O.

**Table 2:** Production of N_2_O by strain JAM1 and the JAM1Δ*narG1narG2* double mutant. Concentrations of ${\mathrm{NO}}_{3}^{-}$, ${\mathrm{NO}}_{2}^{-}$ and N_2_O were measured after 96 h (OD_600nm_ ∼ 1.2) of incubation in strain JAM1 and JAM1Δ*narG1narG2* cultured under oxic conditions with (A) 16.8 mg-N vial^−1^ ${\mathrm{NO}}_{3}^{-}$ added at T0h or (B) 4.7 mg-N vial^−1^ ${\mathrm{NO}}_{2}^{-}$ added at T24h. The results are derived from triplicate cultures. Data represent mean values (SD) (*n* = 3).

Strain	Conditions	${\mathrm{NO}}_{3}^{-}$ (mg-N vial^−1^)	${\mathrm{NO}}_{2}^{-}$ (mg-N vial^−1^)	N_2_O (mg-N vial^-1^)
JAM1	A	0.17 ± 0.06	16.6 ± 0.7	0.14 ± 0.01
JAM1Δ*narG1narG2*	A	17.1 ± 0.1	0.22 ± 0.22	0.004 ± 0.002
JAM1	B	0	4.25 ± 0.09	0.11 ± 0.03
JAM1Δ*narG1narG2*	B	0	4.87 ± 0.39	0.18 ± 0.02

The influence of ${\mathrm{NO}}_{2}^{-}$ was also tested. As the toxicity of ${\mathrm{NO}}_{2}^{-}$ has been attested from 0.36 mM (0.2 mg-N vial^−1^) (Auclair et al., 2010), strain JAM1 and the mutant were cultured without ${\mathrm{NO}}_{3}^{-}$ under oxic conditions to allow for biomass growth. After 24 h, 4.7 mg-N vial^−1^ NO${}_{2}^{-}$ was added to the cultures and was incubated for another 72 h. Strain JAM1 and the mutant produced 0.11 mg-N vial^−1^ and 0.18 mg-N vial^−1^ of N_2_O, respectively, reflecting N_2_O concentrations produced by strain JAM1 under oxic conditions with ${\mathrm{NO}}_{3}^{-}$ (Table 2). Our results show that ${\mathrm{NO}}_{2}^{-}$ and not ${\mathrm{NO}}_{3}^{-}$ is directly involved in N_2_O production, and the Nar systems are not involved in N_2_O production via NO.

### Relative expression levels of denitrification genes in *M. nitratireducenticrescens* JAM1

We assessed whether variations in the expression levels of denitrification genes correlate with the N_2_O accumulation and consumption cycles of strain JAM1 cultures. Strain JAM1 was cultured in NH_4_Cl-free medium with 22 mg-N vial^−1^ ${\mathrm{NO}}_{3}^{-}$ under anoxic conditions. RNA was extracted from cells harvested over four different phases (Fig. 4): (1) at T0 for the pre-cultures (oxic cultures with no ${\mathrm{NO}}_{3}^{-}$), (2) during the growth phase with ${\mathrm{NO}}_{3}^{-}$ reduction and no N_2_O accumulation, (3) during the N_2_O accumulation phase, and (4) during the N_2_O consumption phase. The transcript levels of c*norB1*, c*norB2* and *nosZ,* which encode the catalytic subunits of the corresponding NO and N_2_O reductases, and *nnrS* and *norR*, were measured by RT-qPCR. *nnrS* and *norR* encode NO-sensitive regulators and were used as an indicator of the presence of NO in the cultures. Because the assimilatory nitrate reductase is involved in the re-routing of ${\mathrm{NO}}_{3}^{-}$ to the biomass, RT-qPCR assays were also performed on the gene encoding this reductase (named here *nr*). The expression levels were calculated relative to the transcript levels measured during the preculture phase (set to one) (Fig. 6).

The relative *cnorB1* transcript levels showed an 18.5-fold increase during the growth phase. *cnorB1* expression was still upregulated during the N_2_O accumulation and consumption phases (5.5 and 6.9-fold increases, respectively). The relative *cnorB2* transcript levels had a 1.6-fold increase during the growth phase. These levels returned nearly to the same levels of those in the preculture phase. Significant increases (5.5- and 6.0-fold) of the relative expression levels of *nnrS* were observed in the N_2_O accumulation and consumption phases. *norR* was upregulated (2.3-fold increase) during the growth phase. The *nosZ* expression levels had a 5.4-fold increase during the growth phase relative to the preculture phase, and decreased to the preculture levels during the N_2_O accumulation phase. No significant difference was observed in the relative transcript levels of the *nr* gene between all phases.

## Discussion

Our results show that *M. nitratireducenticrescens* JAM1 can consume NO and N_2_O via the mechanism of reduction of NO to N_2_O and then to N_2_ as predicted by the genome sequence (Fig. S1) (Villeneuve et al., 2013; Mauffrey, Martineau & Villemur, 2015). The N_2_O-amended cultures yielded equivalent biomass results to those of the NO${}_{3}^{-}$-amended cultures as predicted by the respiratory electron transport chains of the denitrification pathway (Simon, 2011). Therefore, in addition of reducing ${\mathrm{NO}}_{3}^{-}$, strain JAM1 has another respiratory capacity under anoxic conditions by reducing N_2_O for its growth.

Although denitrification is generally an anaerobic process, there are cases where it occurs under oxic conditions (Otani, Hasegawa & Hanaki, 2004). As observed with ${\mathrm{NO}}_{3}^{-}$ reduction, NO and N_2_O reduction can occur under oxic conditions, reinforcing the lack of a functional oxygen regulation response in strain JAM1 (Mauffrey, Martineau & Villemur, 2015). However, there is little benefit to this consumption, as N_2_O or ${\mathrm{NO}}_{3}^{-}$ amended cultures have a similar growth pattern than the cultures with only oxygen as terminal electron acceptor. The methylamine-utilizing bacterium *Methylotenera mobilis* strain JLW8 also showed denitrifying activities under oxic conditions (Kalyuhznaya et al., 2009). This freshwater bacterium has an incomplete denitrification pathway with gene clusters encoding a periplasmic NapA-type nitrate reductase, NirK and cNor. Although no growth were recorded in methylamine-amended culture supplemented with ${\mathrm{NO}}_{3}^{-}$ under oxic conditions, growth occurred in methanol-amended cultures with reduction of ${\mathrm{NO}}_{3}^{-}$ to N_2_O. Contrary to *M. nitratireducenticrescens* JAM1, *Methylotenera mobilis* JLW8 cannot grow under anoxic conditions with ${\mathrm{NO}}_{3}^{-}$ (Kalyuzhnaya et al., 2006; Mustakhimov et al., 2013). Denitrification enzymes were showed to be active under anoxic conditions but oxygen is required for strain JLW8 growth (Kalyuhznaya et al., 2009).

N_2_O production was observed in NO${}_{3}^{-}$-amended cultures either under oxic or anoxic conditions when ${\mathrm{NO}}_{2}^{-}$ was accumulating. This production represented up to 6% of N-input in the anoxic cultures, and ${\mathrm{NO}}_{2}^{-}$ was shown to be the key element of this production process. Because we showed that the NO reductase activities were carried out in strain JAM1 cultures, the N_2_O could originate from NO production despite the absence of gene encoding NirS or NirK. Intermediate NO creates problems as this molecule is highly toxic to microorganisms, inducing nitrosative stress in cells (Poole, 2005). Reducing NO is a key step in denitrification and is closely regulated by various sensors and regulators. NnrS and NorR are involved in cell defense against nitrosative stress and are positively regulated by the presence of NO (Stern et al., 2013; Stern et al., 2012; Bartnikas et al., 2002). Therefore, the expression of *nnrS* and *norR* reflects NO concentrations in a medium and was used as a marker of NO presence. The upregulation of the expression of *norR* during the growth phase strongly suggest that NO is produced during this phase. This correlates with higher expressions of both c*norB*, and *nosZ* during the growth phase, which can be regulated by NO-sensitive regulators such as NorR (Spiro, 2012). *nnrS* is upregulated during the N_2_O accumulation and consumption phases, which suggests that NO is still generated during these phases. This upregulation can be linked to the decrease of the relative transcript levels of both *cnorB*, and of *nosZ* and *norR*, but also to the highest level of ${\mathrm{NO}}_{2}^{-}$ concentrations in the culture medium. It is therefore possible that NO is not reduced quickly enough in the cells by cNorB and strain JAM1 must rely on another mechanism, such as NnrS, to protect itself from NO toxicity. Stern et al. (2013) suggest that NnrS does not remove NO directly, but protects cells against the formation of iron-NO complexes, which are inhibitory to iron-sulfur cluster proteins. Moreover, Vaccaro et al. (2016) proposed that NnrS senses NO and signals to cytoplasmic transcription factors or Fe-S cluster repair proteins.

Other ${\mathrm{NO}}_{3}^{-}$ respiring bacteria that lack NirK or NirS have been shown to be N_2_O producers (Bleakley & Tiedje, 1982; Smith & Zimmerman, 1981; Sun, Vos & Heylen, 2016). For instance, *Bacillus vireti* contains three denitrification reductases (Nar, qCu_A_Nor, N_2_OR) and lacks, like *M. nitratireducenticrescens* JAM1, gene encoding NirK or NirS (Mania et al., 2014). This bacterium also produces NO and N_2_O in anaerobic, ${\mathrm{NO}}_{3}^{-}$-amended TSB cultures during ${\mathrm{NO}}_{2}^{-}$ accumulation. NO was shown to originate from chemical decomposition of ${\mathrm{NO}}_{2}^{-}$ (Schreiber et al., 2012) and from an unknown biotic reaction. In our study, the abiotic control of the *Methylophaga* 1403 medium amended with ${\mathrm{NO}}_{3}^{-}$ did not show significant N_2_O production. Also, the abiotic reaction between the ferrous ion in the medium and ${\mathrm{NO}}_{2}^{-}$ generated negligible amount of N_2_O. Furthermore, no N_2_O was detected in this medium inoculated with autoclaved biomass (Fig. 5). These results rule out abiotic reactions as the source of the total amount of N_2_O produced in strain JAM1 cultures. The possible biotic source of NO in absence of NirS or NirK has been studied in *Escherichia coli* (see review by Vine & Cole, 2011). There are supporting evidence that NO is generated in *E. coli* as a side product during ${\mathrm{NO}}_{2}^{-}$ reduction (i) by the cytoplasmic, NADH-dependent nitrite reductase (NirBD), (ii) by the nitrite reductase NrfAB, and (iii) by NarGHI. Vine, Purewal & Cole (2011) showed, with mutants defective in these reductases, that NarGHI is the major enzyme responsible of NO production. However, a small production of NO was still occurring in *narG* mutant, suggesting the involvement of another molybdoprotein. In *M. nitratireducenticrescens* JAM1, the double-knockout mutant JAM1Δ*narG1narG2*, which lacks the two dissimilatory ${\mathrm{NO}}_{3}^{-}$reductases, was still able to produce N_2_O under oxic conditions at the same level of the wild type when ${\mathrm{NO}}_{2}^{-}$ was added to the cultures. These results suggest the two Nar systems are not involved in NO production. The genome of strain JAM1 did not reveal gene encoding NrfAB, but contain a gene cluster encoding a cytoplasmic, NADH-dependent nitrite reductase (CP003390.3; Q7A_2620 and Q7A_2621), which may be the source of NO (Fig. S1). In the latter case, this could be verified by generating a knockout mutant of this gene.

The significance of maintaining an incomplete pathway by *M. nitratireducenticrescens* JAM1 is unclear and may depend upon the original habitat and environment, here the denitrifying biofilm. While *M. nitratireducenticrescens* JAM1 serves as an important actor among the microbial community of the marine biofilm in performing optimal denitrifying activities (Labbé et al., 2003; Labbé, Parent & Villemur, 2003), it was thought to participate uniquely in the reduction of ${\mathrm{NO}}_{3}^{-}$ to ${\mathrm{NO}}_{2}^{-}$. It was previously proposed that ${\mathrm{NO}}_{2}^{-}$ reduction to N_2_ is carried out by *Hyphomicrobium nitrativorans* NL23, the second most represented bacterium in the biofilm (Labbé et al., 2007; Auclair, Parent & Villemur, 2012). Its capacity to reduce NO and N_2_O and to grow on N_2_O suggests that *M. nitratireducenticrescens* JAM1 may participate in the reduction of NO and N_2_O during denitrification in the biofilm. Although our culture assays were performed with high levels of ${\mathrm{NO}}_{3}^{-}$ (37 mM), which rarely exceeds a value of 0.7 mM in natural environments (Yeats, 1990), similar levels can be reached in closed-circuit systems like the seawater aquarium tank located in the Montreal Biodome, where ${\mathrm{NO}}_{3}^{-}$ levels reached up to 14 mM (Parent & Morin, 2000). Rissanen et al. (2016) also observed the combination of *Methylophaga* spp. and *Hyphomicrobium* spp. in the fluidized-bed type denitrification reactors treating the recirculating seawater of the public fish aquarium SEA LIFE at Helsinki, Finland. Although this study provided no indication of the denitrification pathway in these *Methylophaga* and *Hyphomicrobium*, it reinforces the importance of the natural combination of these two genera in marine denitrification environment.

## Conclusions

*M. nitratireducenticrescens* JAM1 is one of few isolated marine methylotrophic bacterial strains to exhibit anaerobic respiratory capacities by reducing ${\mathrm{NO}}_{3}^{-}$ to ${\mathrm{NO}}_{2}^{-}$ and, as reported here, by reducing N_2_O to N_2_. It can also generate N_2_O via NO by an unknown biotic system. Very few marine denitrifying bacteria have been isolated from recirculating marine systems (Borges et al., 2008; Foesel, Drake & Schramm, 2011; Zheng et al., 2012; Zheng et al., 2011). No previous studies have generated genetic information related gene arrangement or expression on these bacteria. Based on substantial data accumulated on the genome, gene arrangement and gene expression of denitrification and on methylotrophy, *M. nitratireducenticrescens* JAM1 can serve as a model for studying such activities in marine environments. Finally, our results enable a better understanding of the ecophysiological role of *M. nitratireducenticrescens* JAM1 in the original biofilm developed in the denitrification reactor of a closed-circuit marine aquarium.

##  Supplemental Information

10.7717/peerj.4098/supp-1Figure S1Schematic of the denitrification pathway and the ammonium pathways in *Methylophaga nitratireducenticrescens* JAM1Functions are based on the annotations of strain JAM1 genome (CP003390.3). Numbers are locus tag of the corresponding gene preceding by Q7A_. TCA, tricarboxylic acid cycle; GS, glutamine synthase; GlnA, Glutamine synthetase; GOGAT, Glutamate dehydrogenase; trp, transporter; deh, dehydrogenase; MDH, methanol dehydrogenase; Fdh, formate dehydrogenase; Cyt, cytochrome; Q, quinolone; Fae, formaldehyde-activating enzyme, HPS, 3-hexulose-6-phosphate synthase; ${\mathrm{NO}}_{3}^{-}$ red and ${\mathrm{NO}}_{2}^{-}$ red, assimilatory nitrate reductase (NR) and NADH-dependent nitrite reductase; NarK1, nitrate/H+ symporter; NarK2, Nitrate/nitrite antiporter; NarK12f, fused NarK1-NarK2 transporter; NarXL, two-component nitrate/nitrite sensor system; NO diox, dioxygenase. Dash lines represent the putative origin of NO from the NADH-dependent nitrite reductase or from an unknown (?) molybdoprotein (Vine & Cole, 2011), and the putative role of NnrS (Vaccaro et al., 2016).

10.7717/peerj.4098/supp-2 Data S1 Raw data

## Additional Information and Declarations

### Data Availability

The following information was supplied regarding data availability: The raw data was provided as a  Supplemental File.
