# Supplementary material for: Denitrifying metabolism of the methylotrophic marine bacterium Methylophaga nitratireducenticrescens strain JAM1
Source: PeerJ. 2017 Nov 28;5:e4098. doi: 10.7717/peerj.4098 (PMC5710167; doi:10.7717/peerj.4098)
Supplement: Figure S1 — Functions are based on the annotations of strain JAM1 genome (CP003390.3). Numbers are locus tag of the corresponding gene preceding by Q7A_. TCA, tricarboxylic acid cycle; GS, glutamine synthase; GlnA, Glutamine synthetase; GOGAT, Glutamate dehydrogenase; trp, transporter; deh, dehydrogenase; MDH, methanol dehydrogenase; Fdh, formate dehydrogenase; Cyt, cytochrome; Q, quinolone; Fae, formaldehyde-activating enzyme, HPS, 3-hexulose-6-phosphate synthase; ${\mathrm{NO}}_{3}^{-}$NO3− red and ${\mathrm{NO}}_{2}^{-}$NO2− red, assimilatory nitrate reductase (NR) and NADH-dependent nitrite reductase; NarK1, nitrate/H+ symporter; NarK2, Nitrate/nitrite antiporter; NarK12f, fused NarK1-NarK2 transporter; NarXL, two-component nitrate/nitrite sensor system; NO diox, dioxygenase. Dash lines represent the putative origin of NO from the NADH-dependent nitrite reductase or from an unknown (?) molybdoprotein (Vine & Cole, 2011), and the putative role of NnrS (Vaccaro et al., 2016). [file peerj-05-4098-s001.pdf]

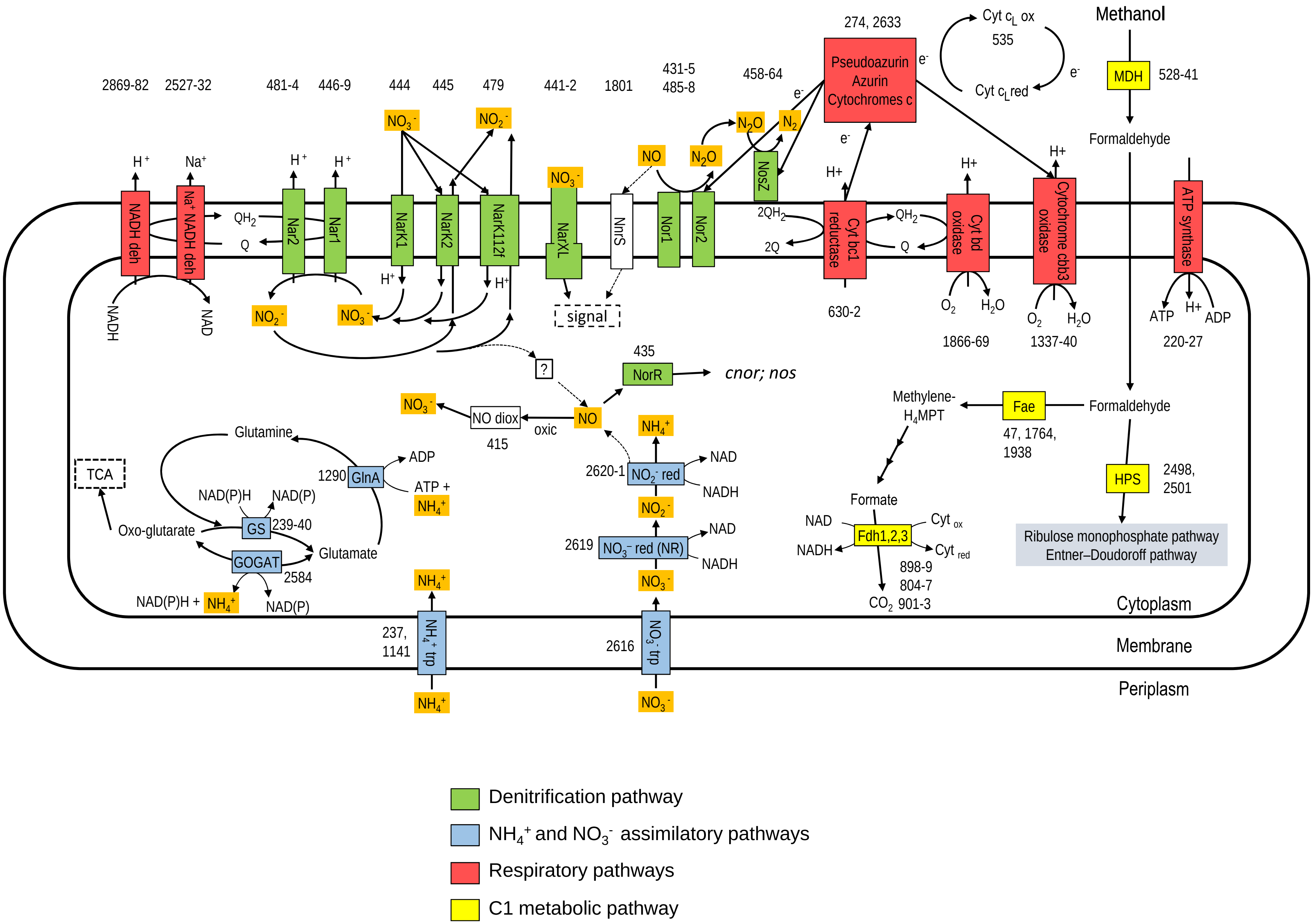

Methanol
MDH
528-41
Formaldehyde
Fae
47, 1764, 1938
Methylene-H4MPT
Formate
Fdh1,2,3
NAD
NADH
Cyt ox
Cyt red
CO2
898-9
804-7
901-3
HPS
2498, 2501
Ribulose monophosphate pathway
Entner–Doudoroff pathway
274, 2633
Pseudoazurin
Azurin
Cytochromes c
Cyt cL ox
535
Cyt cL red
e-
2869-82
2527-32
481-4
446-9
444
445
479
441-2
1801
431-5
485-8
458-64
NADH deh
Na+ NADH deh
Nar2
Nar1
NarK1
NarK2
NarK1I2f
NarXL
NsrS
Nor1
Nor2
NosZ
Cyt bc1 reductase
Cyt bd oxidase
Cytochrome cbb3 oxidase
ATP synthase
H+
Na+
QH2
Q
2QH2
2Q
NO3-
NO2-
NO
N2O
N2
NADH
NAD
O2
H2O
ATP
ADP
630-2
1866-69
1337-40
220-27
signal
?
435
NorR
cnor; nos
NO diox
415
oxic
2620-1
NO2- red
2619
NO3- red (NR)
NH4+
TCA
Glutamine
GlnA
1290
ATP +
NAD(P)H
NAD(P)
GS
239-40
Oxo-glutarate
GOGAT
2584
Glutamate
NAD(P)H +
237, 1141
NH4+ trp
2616
NO3- trp
Cytoplasm
Membrane
Periplasm
Denitrification pathway
NH4+ and NO3- assimilatory pathways
Respiratory pathways
C1 metabolic pathway
